# 25-Hydroxy vitamin D level is associated with sleep disturbances in patients with chronic kidney disease on hemodialysis: a cross-sectional study

**DOI:** 10.3906/sag-1908-87

**Published:** 2020-04-09

**Authors:** Demet YAVUZ, Mehmet Derya DEMIRAĞ, Rahman YAVUZ, Düriye Sila KARAGÖZ ÖZEN, Zeynep Banu RAMAZANOĞLU

**Affiliations:** 1 Clinic of Internal Medicine, Samsun Education and Training Center, University of Health Sciences, Samsun Turkey; 2 Department of Family Medicine, Faculty of Medicine, Ondokuz Mayıs University, Samsun Turkey; 3 Clinic of Infectious Diseases and Clinical Microbiology, Samsun Education and Training Center,University of Health Sciences, Samsun Turkey

**Keywords:** Vitamin D level, sleep disturbances, hemodialysis

## Abstract

**Background/aim:**

Deficient levels of vitamin D are an important factor in the pathogenesis of some neurodegenerative diseases. The aim of this study is to determine the relationship between vitamin D deficiency and depression status and sleep disorders of patients on dialysis.

**Materials and methods:**

In this cross-sectional study, 121 hemodialysis patients were enrolled. All patients had been on hemodialysis for at least six months at the time of the study. Sleep quality and depression status were measured by using specific inventories. All the patients filled out Pittsburg Sleep Quality Index (PSQI) and Beck Depression Inventory (BDI), and gave blood samples. Vitamin D levels were measured for 121 patients, and statistical analysis was done by using SPSS.

**Results:**

Regression analyses demonstrated that low levels of 25(OH)D and high BDI score were independent risk factors for poor sleep quality [ORs were 0.668 (0.566–0.789), 1.080 (1.001–1.164), and 1.080 (1.001–1.164), respectively].

**Conclusion:**

Our results suggest that deficiency of 25(OH)D is an important independent risk factor for poor sleep quality in hemodialysis patients.

## 1. Introduction

Vitamin D is a complex lipophilic molecule which interacts with a specific nuclear receptor and plays a central role in calcium and phosphate homeostasis and musculoskeletal functions [1]. Apart from its well-known metabolic effect, cumulative data have demonstrated that vitamin D has multidirectional effects. For example, nuclear receptors of vitamin D are extensively expressed in some specific brain regions associated with memory and other cognitive processes [2,3]. Hence, deficiency of vitamin D is thought to be an important risk factor in the pathogenesis of cognitive impairment or some neurodegenerative disorders such as Alzheimer’s disease [4]. 

Vitamin D deficiency or insufficiency is very frequent among patients with chronic kidney disease, especially patients with end-stage renal disease (ESRD) [5]. Multiple factors may contribute to vitamin D deficiency in patients with ESRD, such as reduced sun exposure, impaired skin synthesis of endogenous vitamin D depending on poor response to ultraviolet sunlight associated with hyperpigmentation, decreased intake of vitamin D-rich foods, inadequate absorption of vitamin D from the gastrointestinal system, and impaired hepatic conversion of cholecalciferol to calcidiol [6]. Similarly, sleep disturbances are also frequent in patients with ESRD and several factors including age, race, uremia, anemia, hypertension, and malnutrition are thought to be responsible for poor sleep quality in patients with ESRD [7–9]. In addition, both deficiency of vitamin D and sleep disorders in ESRD are also strongly associated with the increased risk of mortality [10,11].

## 2. Materials and methods

### 2.1. Patients and study design

One hundred and twenty-one dialysis patients were enrolled. All patients had been on hemodialysis for at least six months at the time of the study. All patients were treated three times a week with a standard bicarbonate dialysis solution by semisynthetic membranes (dialysis filters surface area from 1.1 to 1.7 m2) and with an average blood flow rate of 300–350 mL/min. In order to measure 25(OH) vitamin D [25(OH)D], all blood samples were obtained between June and August. Serum 25(OH)D concentrations were measured by commercial RIA kit (Immuno-Biological Laboratories, Minneapolis, MN, USA). 

Patients were divided into two groups according to sleep quality. The Pittsburgh Sleep Quality Index (PSQI) was used for assessing the quality of sleep [12]. The patients with a PSQI score of >5 were considered as poor sleepers (22 women, 34 men), while the patients with a PSQI score of ≤5 were considered as good sleepers (44 women, 21 men). Survey forms were completed in face-to-face interviews. This self-administered questionnaire consists of seven components and each component is scored from 0 to 3. Global PSQI score ranges from 0 to 21. The PSQI was validated for the Turkish population by Ağargün et al. [13].

Depression status was evaluated using the Beck Depression Inventory (BDI) [14]. The BDI is an inventory that utilizes the existing symptoms of depression. The BDI consists of 21 questions. The BDI score ranges from 0 to 63. The standard cut-off areas are as follows: 0–9 indicates that a person is not depressed, 10–18 indicates mild depression, 19–29 indicates moderate depression, and 30–63 indicates severe depression [14]. The BDI was validated for the Turkish population by Hisli et al. [15].

Exclusion criteria were as follows: Age <18 years, dementia or mental retardation, psychiatric illness, inability to complete survey forms, history of malignancy, acute or chronic infection, chronic inflammatory illness, history of hospitalization within the last six months, anemia, and parathyroidectomy.

The study protocol was approved by our local scientific ethics committee.

### 2.2. Statistical analysis 

Continuous variables were expressed as mean (SD) or median (min–max) according to the data distribution. Frequencies were expressed as percentage. The chi-square test was used for comparison of the frequencies between two groups. Student’s t*-*test and the Mann–Whitney U test were used for comparison of the continuous variables between groups. Nonparametric Spearman’s rho analysis was used for the correlation analyses. Logistic and linear regression analyses were performed for the determination of independent risk factors for poor sleep quality.

## 3. Results

**Figure 1:**
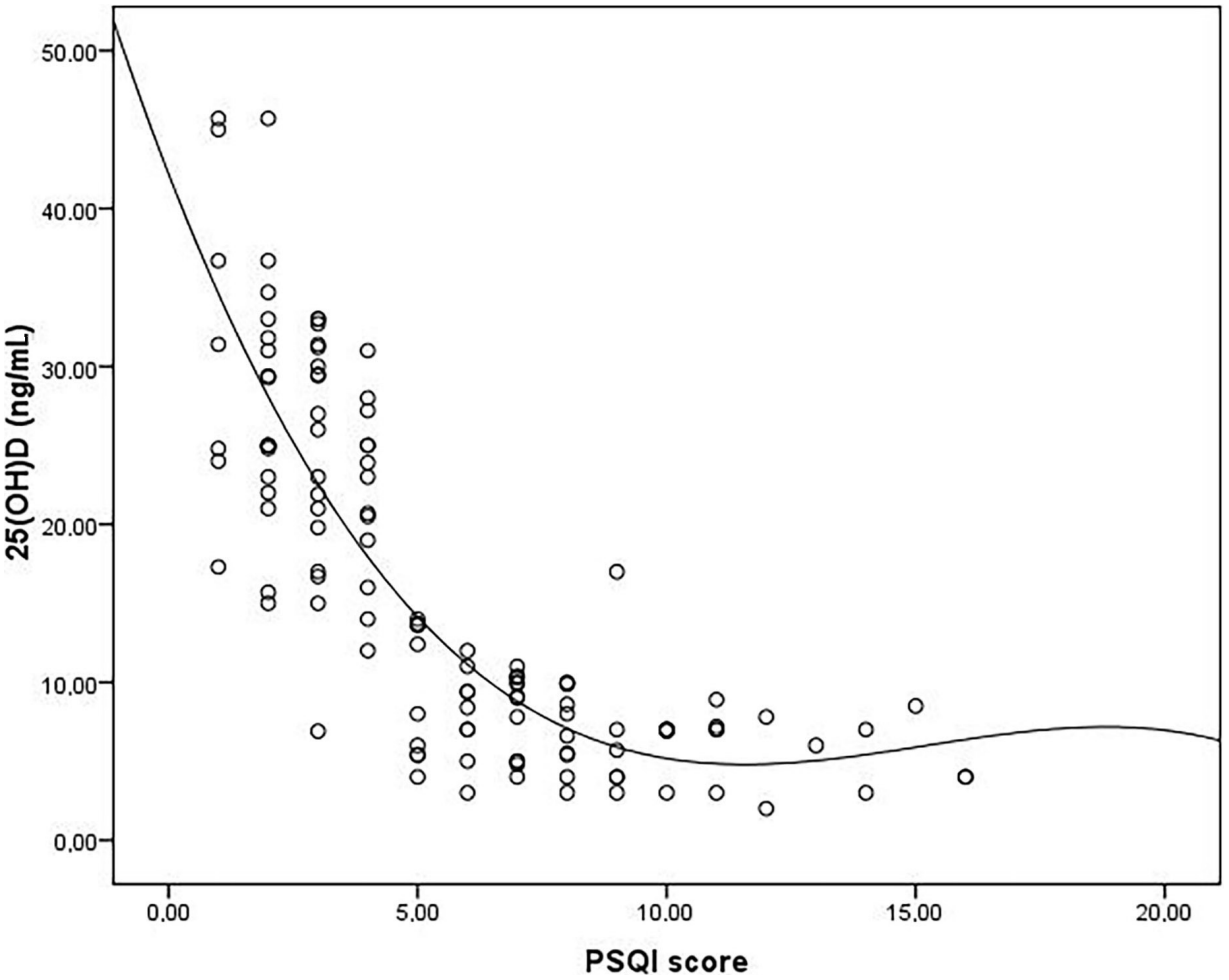
Correlation analysis between 25(OH)D and PSQI score.

**Figure 2:**
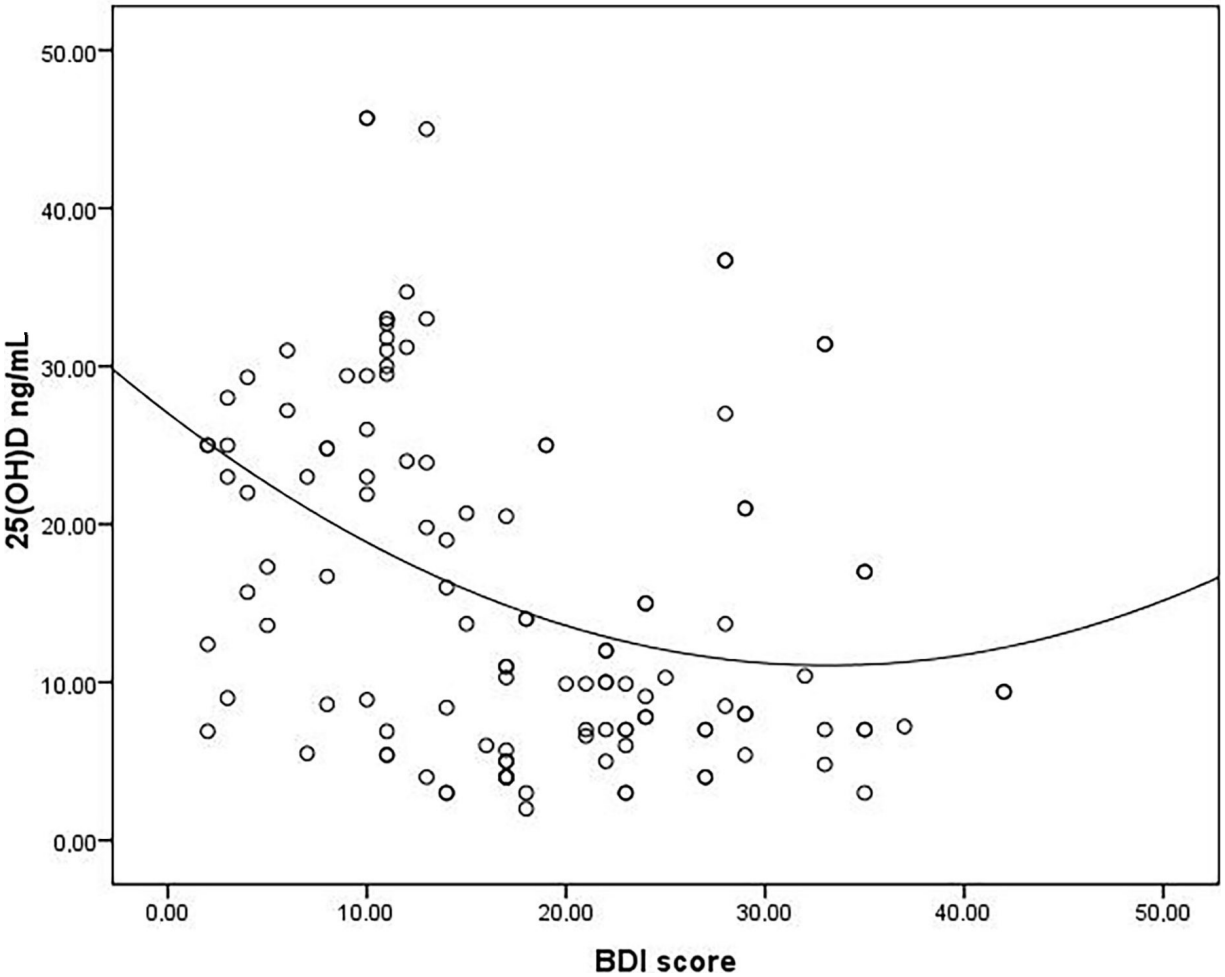
Correlation analysis between 25(OH)D and BDI score.

**Figure 3:**
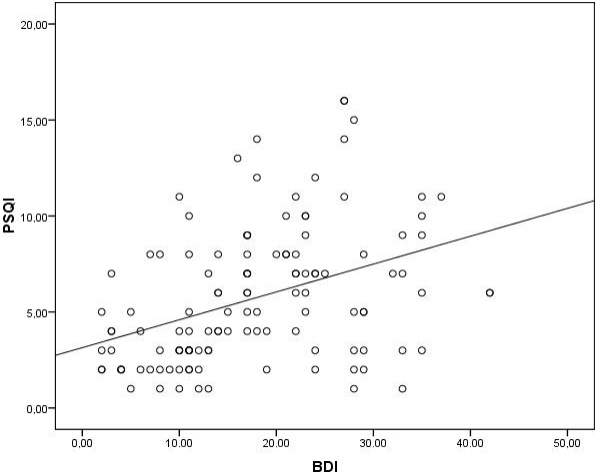
Correlation analysis between PSQI and BDI scores.

Mean age, mean body mass index (BMI), and median duration of dialysis were similar between good and poor sleepers. Mean Kt/V ratio and female/male ratio was significantly higher in good sleepers when compared to the poor sleepers (Table 1). Baseline laboratory parameters were similar between the two groups (Table 2). Median 25(OH)D level was found to be significantly higher in good sleepers when compared to poor sleepers [24 (4–46) vs. 7 (2–17), respectively, P < 0.001]. Median PTH level was found to be significantly higher in good sleepers than poor sleepers [310 (59–1364) vs. 170 (15–1367), respectively, P = 0.013]. Median BDI score was found to be significantly lower in good sleepers than the poor sleepers [11 (2–35) vs. 22 (3–42), respectively, P < 0.001]. The frequency of depressed patients was significantly higher in poor sleepers than good sleepers (64% vs. 29% respectively, P < 0.001). 25(OH)D level was inversely correlated with both PSQI and BDI scores (r = –0.82, P < 0.001 and r = –0.36, P < 0.001, respectively) (Figures 1 and 2). There was a significant positive correlation between PSQI and BDI scores (r = 0.43, P < 0.001) (Figure 3). Logistic regression analyses demonstrated that low levels of 25(OH)D and high BDI score were independent risk factors for poor sleep quality (Table 3). Linear regression analyses also demonstrated that low levels of 25(OH)D and low Kt/V values were independent risk factors for poor sleep quality. 

**Table 1:** Baseline characteristic of the study groups.

	Good sleepers	Poor sleepers	P
Age, mean (SD), years	55 (14)	57 (15)	NS
Sex, F/M	44/21	22/34	<0.01
BMI, mean (SD), kg/m2	25.3 (5.5)	27.1 (4.4)	NS
Duration of dialysis, median (min-max), months	38 (6–216)	25 (6–192)	NS
Kt/V, mean (SD)	1.3 (0.2)	1.4 (0.3)	<0.001

**Table 2:** Laboratory parameters between good and poor sleepers.

Mean (SD)	Good sleepers	Poor sleepers	P
BUN, mg/dL	62.1 (14.8)	64.6 (13.7)	NS
Creatinine, mg/dL	7.8 (1.9)	7.2 (1.7)	NS
Calcium, mg/dL	8.6 (0.6)	8.5 (0.6)	NS
Phosphorus, mg/dL	5.1 (1.3)	4.9 (1.4)	NS
Total cholesterol, mg/dL	151 (38)	161 (39)	NS
LDL, mg/dL	83 (30)	93 (28)	NS
Triglyceride, mg/dL	188 (112)	188 (108)	NS
Albumin, g/dL	3.9 (0.3)	3.8 (0.3)	NS
Hemoglobin, g/dL	11.2 (1.2)	11.3 (1.1)	NS

**Table 3:** Risk factor analysis for the sleep quality.

	Sleep quality				
	Logistic regression		Linear regression		
	OR (95% CI)	P	β	T	P
25(OH)D	0.668 (0.566–0.789)	<0.001	–0.682	–10.756	<0.001
BDI	1.080 (1.001–1.164)	0.047	-	-	NS
Kt/V	-	NS	–0.182	–2.873	0.005

## 4. Discussion 

Sleep disturbances are common problems in hemodialysis patients. Although the exact pathogenetic mechanism of poor sleep in hemodialysis patients still remains unknown, many factors are thought to be responsible for this condition such as age, race, renal function, and some metabolic parameters [7–9]. In the present study, we evaluated the relationship between 25(OH)D levels, sleep disturbance, and depression in ESRD patients. To the best of our knowledge, this is the first report that investigates the effect of 25(OH)D on sleep status in hemodialysis patients.

We found that the levels of 25(OH)D were significantly higher in good sleepers when compared to the poor sleepers. In the medical literature, the relationship between 25(OH)D status and sleep disturbances has been extensively studied in subjects with normal kidney function. In a cross-sectional study, Çakır et al. demonstrated that PSQI scores were significantly higher in patients with vitamin D deficiency when compared to the subjects who had normal levels of vitamin D [16]. In another large cross-sectional study, Bertisch et al. found that vitamin D deficiency was strongly associated with short sleep duration depending on ethnicity [17]. In addition, cross-sectional data from the National Health and Nutrition Examination Surveys (NHANES) 2005–2006 also demonstrated that short sleep duration was significantly associated with levels of 25(OH)D [18].

Depression is an important causal factor for sleep disturbances in patients with chronic renal failure. Hemodialysis and peritoneal dialysis patients who were poor sleepers were found to be more depressed [19,20]. We found that BDI score was significantly lower in good sleepers when compared to the poor sleepers. Our results also demonstrated that there was a significant positive correlation between PSQI and BDI scores. At first sight, these results may thought to be confounding factors for the relationship between 25(OH)D and sleep disturbances, and our subjects with poor sleep were more depressed when compared to the good sleepers. However, our regression analyses demonstrated that a low level of 25(OH)D was an independent risk factor for poor sleep quality. Another important confounding factor for sleep disturbance in hemodialysis patient is the efficacy and adequacy of the hemodialysis. The Kt/V ratio is a way of measuring dialysis adequacy. Hemodialysis patients with sleep disturbances have low Kt/V ratios [21]. In our study, Kt/V ratios and parameters that may affect sleep status such as age, duration of hemodialysis, body mass index, and hemoglobin levels were found to be similar between good and poor sleepers. Although sex distribution and Kt/V were statistically different between groups, we demonstrated that a low level of 25(OH)D was an independent risk factor for poor sleep quality. 

Vitamin D receptors are extensively expressed in the anterior and posterior hypothalamus, midbrain central gray, and the basal forebrain. All of these brain areas appear to coordinate the sleep/wake state [2,3]. On the other hand, many neurotransmitters and neuromodulators such as dopamine, serotonin, norepinephrine, glutamate, and γ-aminobutyric acid are important players in the regulation and maintenance of sleep/wake-dependent changes in neuronal activity and the sleep/wake continuum and dysregulation of these neurochemical systems leads to sleep/wake disorders [22]. The levels of these neurotransmitters in the brain may be modulated by vitamin D [23]. Vitamin D metabolites can also inhibit the synthesis of inducible nitric oxide synthase and increase glutathione levels like a hormone in brain detoxification pathways [24]. All these features of vitamin D may partly explain the role of vitamin D on the pathogenesis of sleep disturbance.

In conclusion, vitamin D deficiency and sleep disturbance are common problems of hemodialysis patients. In addition, both 25(OH)D deficiency [10] and sleep disturbance [11] in chronic kidney disease are two important risk factors associated with mortality. Cholecalciferol therapy may increase serum 25(OH)D levels in patients on maintenance hemodialysis and cholecalciferol supplementation is found to be effective and safe in hemodialysis patients [25,26]. Our results suggest that deficiency of 25(OH)D is an important risk factor for poor sleep quality in hemodialysis patients, and this result is independent of other factors such as sex, age, depression, and efficacy of hemodialysis. All hemodialysis patients who suffer from sleep disturbance should be carefully evaluated for vitamin D status.

## References

[ref0] (2016). Crucial role of vitamin D in the musculoskeletal system. Nutrients.

[ref1] (1992). Vitamin D nuclear binding to neurons of the septal, substriatal and amygdaloid area in the Siberian hamster (Phodopus sungorus) brain. Neuroscience.

[ref2] (1987). 2 vitamin D3 sites of action in the brain. O’Brien LP.

[ref3] (2015). Vitamin D status and its association with gradual decline in cognitive function. Turkish Journal of Medical Sciences.

[ref4] (2004). Vitamin D insufficiency and deficiency in chronic kidney disease. A single center observational study. American Journal of Nephrology.

[ref5] (2012). Ergocalciferol and cholecalciferol in CKD. American Journal of Kidney Diseases.

[ref6] (2006). Sleep behavior disorders in a large cohort of Chinese (Taiwanese) patients maintained by long-term hemodialysis. American Journal of Kidney Diseases.

[ref7] (2008). Sleep disturbances in dialysis patients. Journal of Nephrology.

[ref8] (2003). Quality of sleep and health-related quality of life in haemodialysis patients. Nephrology Dialysis Transplantation.

[ref9] (2016). Low vitamin D and high fibroblast growth factor 23 serum levels associate with infectious and cardiac deaths in the HEMO Study. Journal of American Society Nephrology.

[ref10] (2014). Effects of sleepiness on survival in Japanese hemodialysis patients: J-DOPPS study. Nephron Clinical Practice.

[ref11] (1989). The Pittsburgh Sleep Quality Index: a new instrument for psychiatric practice and research. Psychiatry Research.

[ref12] (1996). Validity and reliability of Pittsburgh Sleep Quality Index. Türk Psikiyatri Dergisi.

[ref13] (1961). An inventory for measuring depression. Archives of General Psychiatry.

[ref14] (1988). Envanteri’nin geçerliliği üzerine bir çalışma. Psikoloji Dergisi.

[ref15] (2015). An evaluation of sleep quality and the prevalence of restless leg syndrome in vitamin D deficiency. Acta Neurologica Belgica.

[ref16] (2015). and sleep duration and continuity: multi-ethnic study of atherosclerosis. Sleep.

[ref17] (2014). Serum nutritional biomarkers and their associations with sleep among US adults in recent national surveys. PLoS One.

[ref18] (2005). Quality of sleep and quality of life in renal transplantation patients. Transplantation Proceedings.

[ref19] (2008). Sleep quality and depression in peritoneal dialysis patients. Renal Failure.

[ref20] (2007). Sleep disturbances in patients on maintenance hemodialysis: role of dialysis shift. Revista da Associação Médica Brasileira.

[ref21] (2016). Sleep pharmacogenetics: personalized sleep-wake therapy. Annual Review of Pharmacology Toxicology.

[ref22] (2014). Neurochemical effects of chronic administration of calcitriol in rats. Nutrients.

[ref23] (1996). Dihydroxyvitamin D3 regulates gamma 1 transpeptidase activity in rat brain. Neuroscience Letters.

[ref24] (2013). Cholecalciferol in haemodialysis patients: a randomized, double-blind, proof-of-concept and safety study. Nephrology Dialysis Transplantation.

